# Variola and Varicella

**Published:** 1902-07-12

**Authors:** 


### VARIOLA AND VARICELLA.

In a paper by Dr. McConnel Wanklyn,[1] who has for some time acted as small-pox referee and medical superintendent of the River Ambulance Service. some useful hints are given as to the differential diagnosis between variola and varicella. An accurate history of the attack is, he says, a rarity, but even an accurate history may be misleading, for cases of varicella in the adult may give a history very like that of small-pox. In regard to general symptoms, he points out that sacral pain and vomiting are uncommon in varicella. What he insists upon most strongly in regard to general symptoms is that in small-pox there is a general muscular flaccidity and tonelessness. The phenomenon is to be noted in all the voluntary muscles. The patient lies like a man prostrate after severe physical exertion, with limbs flaccid and all his muscles relaxed. Especially does this apply to the facial muscles. "In a patient marked by an abundant rash, whose expression is alert, attention keen and mentalisation acute, the diagnosis of small-pox is unlikely. . . . A patient suffering from small-pox in a corresponding degree has a dull, expressionless facies, with a settled aspect of fatigue and inattention."

In regard to the rash it is to be noted that its distribution is a matter of cardinal importance. In the revision of the diagnosis of some 7,000 cases examined during the present epidemic no one criterion has proved more reliable and useful. It is well known that the small-pox eruption is abundant on the face, wrists, and hands, and often on the feet, whereas in comparison it is light upon the trunk. It is also well recognised that the site of election for the rash of varicella is upon the trunk, and that the forearms and hands are lightly affected. What is not so generally known is the constancy and reliability of this phenomenon. It is not uncommon in a severe case of varicella, especially in an adult, for the face to show a dense rash and to be more thickly covered than the back and front of the trunk. But the rash in such a case, as in all cases of varicella, will show a very light relative incidence on the forearms and hands, as well as on the legs and feet. The more distal the rash is upon the extremities the scantier and smaller it is found to be, and in some cases where the lesions are numbered on the face by hundreds the skin of the hands is free from any blemish whatever. Not that varicella is excluded by the occurrence of vesicles on the palms or soles. It is the relative distribution and density of the rash which are to be considered.

As to the character of the rash the words "shotty" and "umbilicated" are but broken reeds to lean upon in diagnosis. In the differential diagnosis of small-pox there are no more fertile causes of error. The true distinction between individual lesions of variola and varicella lies in the depth in the skin at which the lesion is placed. As compared with variola the varicella rash is superficial. This is evident in the early formation of the vesicle and its rapid maturation, the early formation and rapid detachment of the scab, and in the lightness of the scar. The occasional deep ulcers and scarring which occur in varicella commonly result from scratching and the insertion of dirt. The points by which the superficial nature of the lesions in varicella are most easily recognised are the fineness and thinness of the pelicle covering the vesicle, giving to it the familiar translucency. The shape and outline of the vesicle are also characteristic, its position in the skin allowing it often to take on an irregularly oval outline, the long axis of which is parallel to the creases of the skin of the part. The situation where these are seen most commonly and clearly are the folds of the axilla and flank ; the groins and lumbar region also often show similar characteristic lesions. Again the outline is often crenated or irregular, while owing to the more yielding character of the tissues immediately surrounding it the tension is not so high in a varicella as in a variola vesicle. Evacuating the contents by a needle is apt to give rise to erroneous conclusions, for complete evacuation has been noted in several of the cases of variola in which the lesions when two days old were frankly vesicular. A point worth noting is the irregular character of the lesions in varicella on a given area. They may vary in size as well as in shape and stage of development. In a late stage of either disorder the diagnosis rests almost entirely upon the distribution of the scars and such scabs as remain. The diagnosis of small-pox is clinched if the evenly circular disc-like scabs are found, and the brown inspissated remains of pustules in the thick skin of the hands and feet. The differential diagnosis between these diseases does not rest upon any one feature but upon a careful consideration of all the evidence, especial importance being given to the depth in the skin at which the lesions are placed and above all to the distribution of the rash.

[1] Brit. Med. Jour., July 5.

